# Adiponectin receptor-mediated signaling ameliorates cerebral cell damage and regulates the neurogenesis of neural stem cells at high glucose concentrations: an *in vivo* and *in vitro* study

**DOI:** 10.1038/cddis.2015.220

**Published:** 2015-08-06

**Authors:** J Song, S M Kang, E Kim, C-H Kim, H-T Song, J E Lee

**Affiliations:** 1 Department of Anatomy, Yonsei University College of Medicine, Seoul 120-752, South Korea; 2 BK21 Plus Project for Medical Sciences and Brain Research Institute, Yonsei University College of Medicine, Seoul 120-752, South Korea; 3 Department of Psychiatry, Yonsei University College of Medicine, Seoul 120-752, South Korea; 4 Department of Pharmacology, Yonsei University College of Medicine, Seoul 120-752, South Korea; 5 Department of Diagnostic Radiology, Yonsei University College of Medicine, Seoul 120-752, South Korea

## Abstract

In the central nervous system (CNS), hyperglycemia leads to neuronal damage and cognitive decline. Recent research has focused on revealing alterations in the brain in hyperglycemia and finding therapeutic solutions for alleviating the hyperglycemia-induced cognitive dysfunction. Adiponectin is a protein hormone with a major regulatory role in diabetes and obesity; however, its role in the CNS has not been studied yet. Although the presence of adiponectin receptors has been reported in the CNS, adiponectin receptor-mediated signaling in the CNS has not been investigated. In the present study, we investigated adiponectin receptor (AdipoR)-mediated signaling *in vivo* using a high-fat diet and *in vitro* using neural stem cells (NSCs). We showed that AdipoR1 protects cell damage and synaptic dysfunction in the mouse brain in hyperglycemia. At high glucose concentrations *in vitro*, AdipoR1 regulated the survival of NSCs through the p53/p21 pathway and the proliferation- and differentiation-related factors of NSCs via tailless (TLX). Hence, we suggest that further investigations are necessary to understand the cerebral AdipoR1-mediated signaling in hyperglycemic conditions, because the modulation of AdipoR1 might alleviate hyperglycemia-induced neuropathogenesis.

Adiponectin secreted by the adipose tissue^1, 2^ exists in either a full-length or globular form.^3, 4, 5, 6^ Adiponectin can cross the blood–brain barrier, and various forms of adiponectin are found in the cerebrospinal fluid.^7, 8, 9, 10, 11^ Adiponectin exerts its effect by binding to the adiponectin receptor 1 (AdipoR1) and adiponectin receptor 2 (AdipoR2)^12, 13^ that have different affinities for the various circulating adiponectins.^12, 14, 15, 16, 17^ Several studies reported that both receptor subtypes are expressed in the central nervous system (CNS).^7, 12, 18^ As adiponectin modulates insulin sensitivity and inflammation,^19^ its deficiency induces insulin resistance and glucose intolerance in animals fed a high-fat diet (HFD).^19, 20, 21^ In addition, adiponectin can ameliorate the glucose homeostasis and increase insulin sensitivity.^22, 23, 24^ Adiponectin, which is the most well-known adipokine, acts mainly as an anti-inflammatory regulator,^25, 26^ and is associated with the onset of neurological disorders.^27^ In addition, a recent study reported that adiponectin promotes the proliferation of hippocampal neural stem cells (NSCs).^28^ Considering that adiponectin acts by binding to the adiponectin receptors, investigation of the adiponectin receptor-mediated signaling in the brain is crucial to understand the cerebral effects of adiponectin and the underlying cellular mechanisms.

The prevalence of type II diabetes mellitus (DM2) and Alzheimer's disease increases with aging.^29^ According to a cross-sectional study, in people with DM2, the risk of dementia is 2.5 times higher than that in the normal population.^30, 31^ A study performed between 1980 and 2002 suggested that an elevated blood glucose level is associated with a greater risk for dementia in elderly patients with DM2.^32^ In addition, according to a 9-year-long longitudinal cohort study, the risk of developing Alzheimer's disease was 65% higher in people with diabetes than in control subjects.^33^ A community-based cohort study also reported that higher plasma glucose concentrations are associated with an increased risk for dementia, because the higher glucose level has detrimental effects on the brain.^31^ High blood glucose level causes mitochondria-dependent apoptosis,^34, 35, 36^ and aggravates diverse neurological functions.^37, 38^ Inflammation and oxidative stress, which are commonly observed in people with diabetes, inhibit neurogenesis.^39, 40, 41^ Similarly, neurogenesis is decreased in mice and rats with genetically induced type I diabetes.^42, 43^ In addition, diabetic rodents have a decreased proliferation rate of neural progenitors.^43, 44^ Furthermore, several studies suggested that an HFD leads to neuroinflammation, the impairment of synaptic plasticity, and cognitive decline.^45, 46^

Here, we investigated whether AdipoR1-mediated signaling is associated with cell death in the brain of mice on a HFD, and whether high glucose level modifies the proliferation and differentiation capacity of NSCs *in vitro*. Our study provides novel findings about the role of AdipoR1-mediated signaling in hyperglycemia-induced neuropathogenesis.

## Results

### HFD led to cell death in the mouse brain

**Figure 1:**
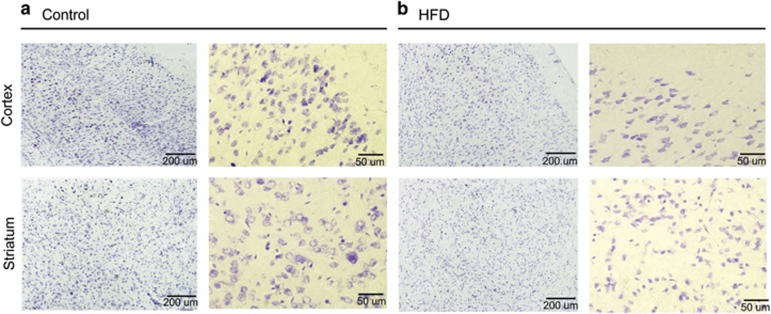
Histological assessment of the brain of mice fed a high-fat diet (HFD) using cresyl violet staining. Cresyl violet staining revealed a slight cell loss in the cortex and striatum of mice fed a HFD (**b**), whereas cells in the control group had more healthy and round cell bodies in the cortex and striatum (**a**). Scale bar: 200 and 50 *μ*m. Control: control group; HFD: high-fat diet group

**Figure 2:**
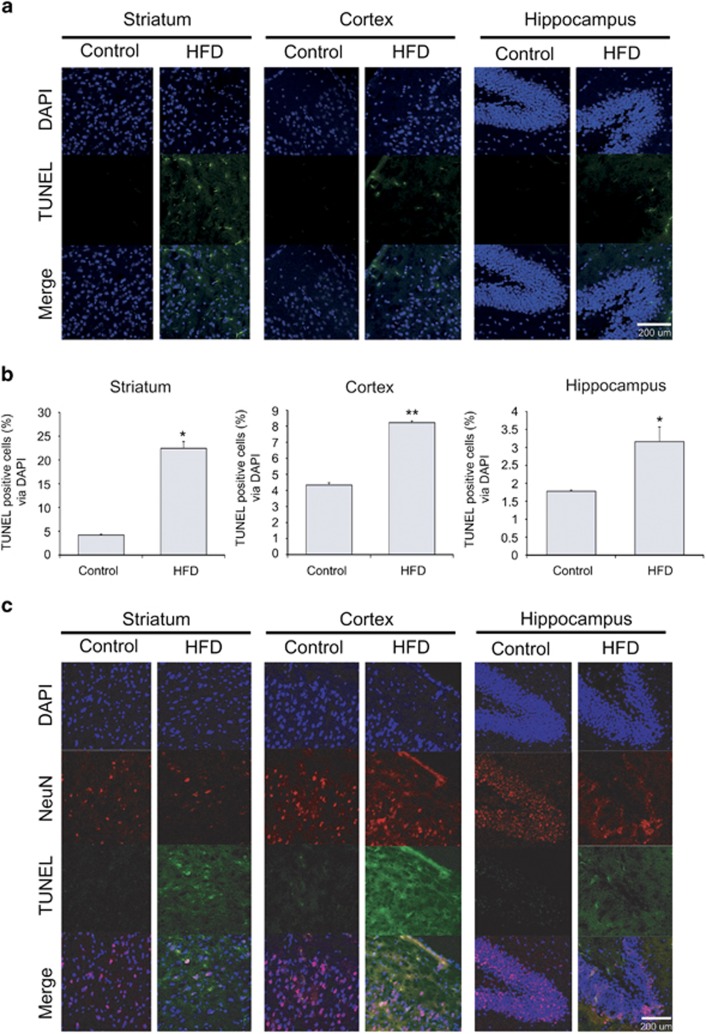
Measurement of cell damage using the TUNEL assay. (**a**) TUNEL-positive signal (green), which indicates cells with DNA damage, was increased in the brain of mice fed a high-fat diet (HFD). (**b**) Similarly, TUNEL signal was increased in the cortex, striatum, and hippocampus in the HFD group compared with that in the control group. (**c**) NeuN (red)-positive cells were decreased in the HFD brain striatum and hippocampus. TUNEL (green)- and NeuN (red)-positive signals were also increased in the HFD brain compared with the control group. Scale bar: 200 *μ*m. Control: control group, HFD: high-fat diet group. 4',6-diamidino-2-phenylindole (DAPI): blue; TdT-mediated dUTP nick end labeling (TUNEL): green; NeuN: red. *P<0.05, **P<0.001

To assess the morphological alterations of neurons in mice fed a HFD, we performed cresyl violet staining in the cortex and striatum (Figure 1). In the control group, healthy round cells were observed in these areas (Figure 1a). In the HFD group, thin small cell bodies and damaged brain tissue were visible in the cortex and striatum (Figure 1b). To identify whether apoptotic cells were present in the brain tissues of mice fed a HFD, we used the TdT-mediated dUTP nick end labeling (TUNEL) assay^47^ (Figure 2). More TUNEL-positive cells were observed in the cortex, striatum, and hippocampus of mice fed a HFD than those in the brain regions of the control mice (Figure 2). Our data suggest that HFD damages cells in the cortex, striatum, and hippocampus (Figures 2a and b). In addition, we assume colocalization with the NeuN, known as the marker of neuron in TUNEL-positive cells (Figure 2c). Figure 2c indicates that neuronal cells may be damaged in HFD brain in comparison with the normal brain.

### HFD decreased the expression of PSD95 and DCX in the mouse brain

**Figure 3:**
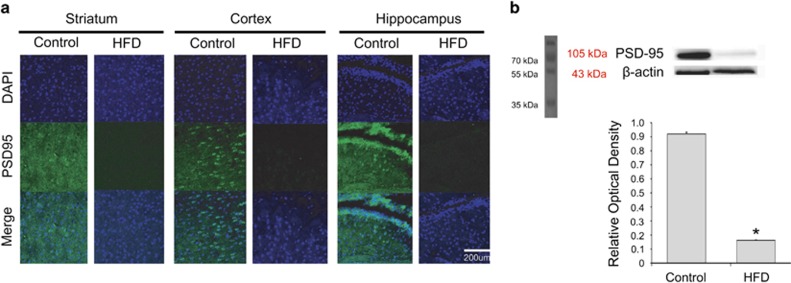
Measurement of PSD95 expression in the brain of mice fed a high-fat diet (HFD). (**a**) Immunohistochemical experiments revealed that the PSD95-positive immunostaining (green) was attenuated in the brain of mice fed a HFD. In addition, PSD95 immunoreactivity was reduced in the striatum, cortex, and hippocampus of the HFD group compared with those of the control group. Scale bar: 200 *μ*m. 4',6-diamidino-2-phenylindole (DAPI): blue; postsynaptic density protein 95 (PSD95): green. (**b**) The protein level of PSD95 was significantly reduced in the HFD group compared with that in the control group. *β*-Actin was used as internal control. Data are expressed as mean±S.E.M., and each experiment included three repeats per condition. Differences were considered significant at *P<0.05. Control: control group; HFD: high-fat diet group

**Figure 4:**
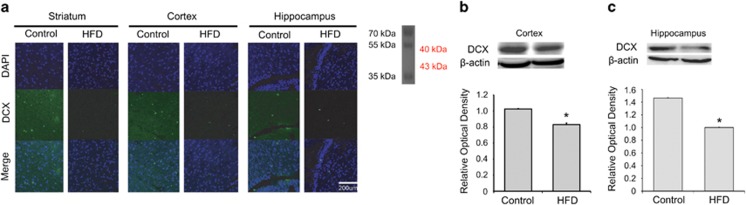
Measurement of DCX expression in the brain of mice fed a high-fat diet (HFD). (**a**) Immunohistochemical experiments revealed that the DCX-immunopositive signal (green) was reduced in the brain of mice fed a HFD. In addition, DCX immunoreactivity was decreased in the striatum, cortex, and hippocampus of the HFD group compared with those of the control group. Scale bar: 200 *μ*m. 4',6-diamidino-2-phenylindole (DAPI): blue; doublecortin (DCX): green. (**b**) The protein level of DCX was significantly reduced in the HFD brain cortex compared with that in the control group. *β*-Actin was used as internal control. Data are expressed as mean±S.E.M., and each experiment included four repeats per condition. Differences were considered significant at *P<0.05. (**c**) The protein level of DCX was a little decreased in the HFD brain hippocampus in comparison with the control group. *β*-Actin was used as internal control. Data are expressed as mean±S.E.M., and each experiment included four repeats per condition. Differences were considered significant at *P<0.05. Control: control group; HFD: high-fat diet group

To determine the alterations caused by HFD in a protein regulating synaptic plasticity and in a neuronal microtubule-associated protein, we performed immunohistochemistry using the antibodies specific for postsynaptic density protein 95 (PSD95), a protein in the postsynaptic density (Figure 3), and for doublecortin (DCX), a microtubule-associated protein involved in neuronal migration (Figure 4). The immunoreactivity for PSD95 synaptic density protein was reduced in the mice fed a HFD compared with that in the control group (Figure 3a). This result indicates that HFD suppresses PSD95 expression in the striatum, cortex, and hippocampus. Similarly, the protein level of PSD95 was reduced in the HFD group (Figure 3b). To examine the expression of the neuronal microtubule protein DCX, we performed immunohistochemical (Figure 4a) and western blot (Figures 4b and c) analyses. DCX immunoreactivity was reduced in the brain of mice fed a HFD compared with that of the control group (Figure 4a). Western blot revealed a similar decrease in the protein level of DCX in cortex (Figure 4b) and in hippocampus (Figure 4c). Our results suggest that HFD damaged the synaptic plasticity and reduced the amount of immature neuronal NSCs in the brain.

### HFD attenuated the expression of TLX in the mouse brain

**Figure 5:**
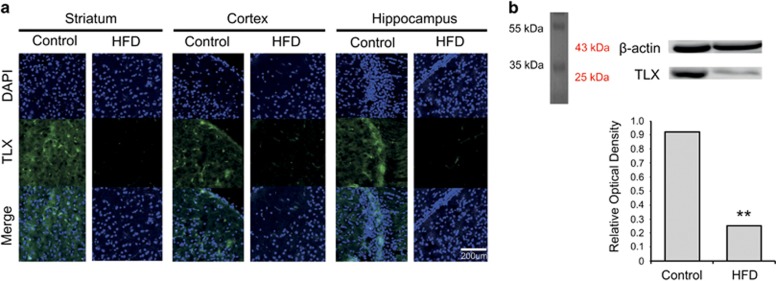
Measurement of TLX expression in the brain of mice fed a high-fat diet (HFD). (**a**) Immunohistochemical experiments revealed that the TLX-positive immunostaining (green) was attenuated in the brain of mice fed a HFD. In addition, TLX immunoreactivity was reduced in the striatum, cortex, and hippocampus of the HFD group compared with those in the control group. Scale bar: 200 *μ*m. 4',6-diamidino-2-phenylindole (DAPI): blue; TLX: green. (**b**) The protein level of TLX was reduced in the HFD group compared with that in the control group. *β*-Actin was used as internal control. Data were expressed as mean±S.E.M., and each experiment included four repeats per condition. Differences were considered significant at **P<0.001. Control: control group; HFD: high-fat diet group

To determine alterations in a differentiation and proliferation-related transcription factor in the brain, we checked the expression of tailless (TLX) in mice fed a HFD (Figure 5). TLX immunoreactivity was reduced in the brain of mice fed a HFD compared with that of the control animals (Figure 5a). The protein level of TLX in the striatum, cortex, and hippocampus of mice fed a HFD was attenuated compared with that of the control mice (Figure 5b). As several studies demonstrated that the TLX transcription factor is related to self-renewal and neurogenesis of the NSCs,^48, 49, 50^ our findings suggest that HFD reduces TLX expression in the brain.

### HFD reduced AdipoR1 expression

**Figure 6:**
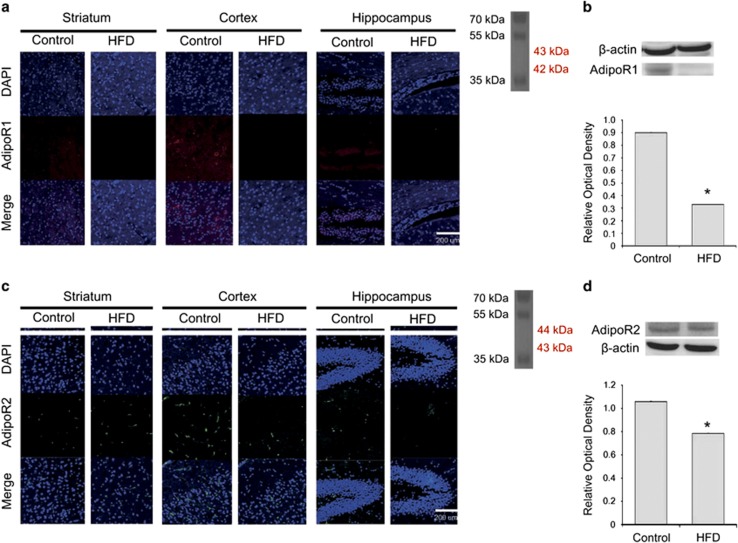
Measurement of AdipoR1 and AdipoR2 expression in the brain of mice fed a high-fat diet (HFD). (**a**) Immunohistochemical experiments revealed that the AdipoR1-positive immunolabeling (red) was attenuated in the brain of mice fed a HFD. In addition, AdipoR1 immunoreactivity was reduced in the striatum, cortex, and hippocampus of the HFD group compared with those of the control group. Scale bar: 200 *μ*m. 4',6-diamidino-2-phenylindole (DAPI): blue; AdipoR1: red. (**b**) Western blotting experiments showed that the relative protein level of AdipoR1 was reduced in the HFD group compared with that in the control group. *β*-Actin was used as internal control. Data are expressed as mean±S.E.M., and each experiment included three repeats per condition. Differences were considered significant at *P<0.05. (**c**) Immunohistochemical experiments revealed that the AdipoR2-positive immunolabeling (green) was attenuated in the brain of mice fed a HFD. In addition, AdipoR2 immunoreactivity was reduced in the striatum, cortex, and hippocampus of the HFD group compared to those of the control group. Scale bar: 200 *μ*m. 4',6-diamidino-2-phenylindole (DAPI): blue; AdipoR2: green. (**d**) Western blotting experiments showed that the relative protein level of AdipoR2 was slightly reduced in the HFD group compared with that in the control group. *β*-Actin was used as internal control. Data are expressed as mean±S.E.M., and each experiment included three repeats per condition. Differences were considered significant at *P<0.05. Control: control group; HFD: high-fat diet group

To assess the expression of AdipoR1 and AdipoR2 in the brain of mice fed a HFD, we performed immunohistochemical analysis (Figures 6a and c) and western blotting (Figures 6b and d) using specific antibodies. Immunoreactivity for AdipoR1 in brain of mice fed a HFD was decreased compared with that in the control group (Figure 6a). Western blot experiments showed that the protein level of AdipoR1 in the striatum, cortex, and hippocampus was reduced in the HFD group (Figure 6b). Immunoreactivity for AdipoR2 in HFD brain were reduced compared with the control group (Figure 6c). The protein levels of AdipoR2 in HFD brain were slightly attenuated in comparison with the control group brain (Figure 6d). Our findings suggest that HFD decreases AdipoR expression, and attenuates the AdipoR-mediated signaling in the brain.

### Adiponectin maintains the neurosphere size of NSCs at high glucose concentrations *in vitro*

**Figure 7:**
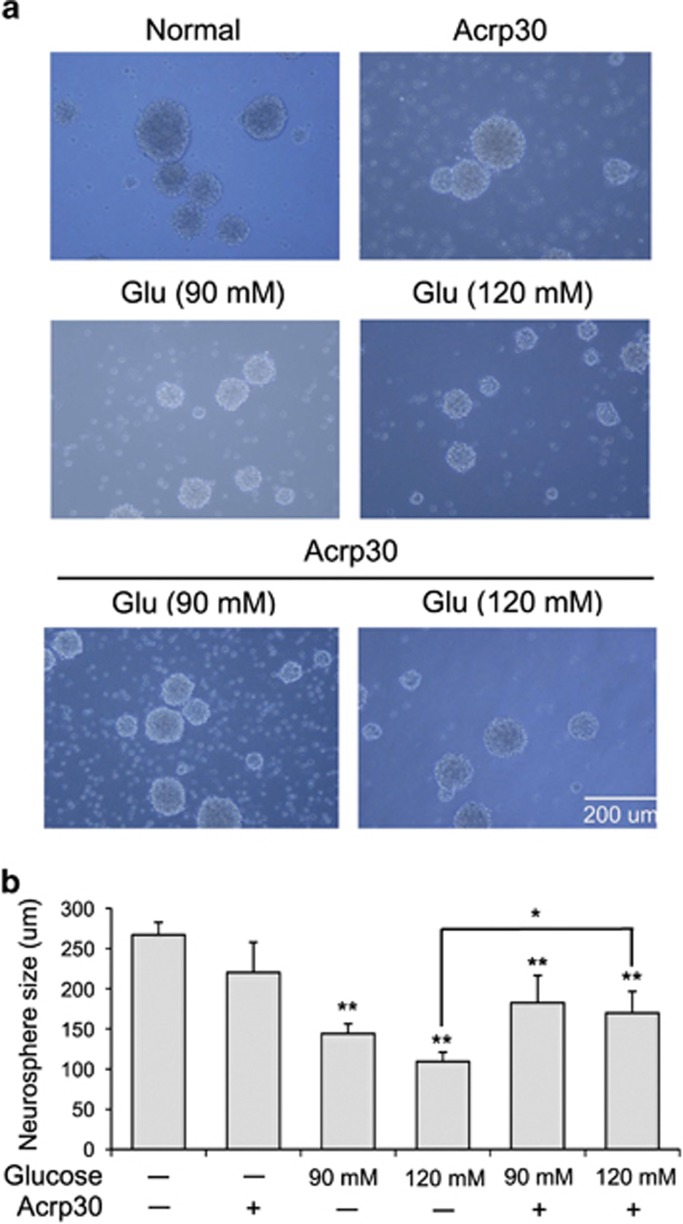
Measurement of neurosphere size at high glucose concentration. (**a**) Neurospheres were observed using bright-field microscopy, and neurosphere size was measured using the Image J (Madison, Wisconsin, USA). Size of neurospheres was identical in the Acrp30 treatment and the control groups. Glucose treatment reduced the neurosphere size compared with the control group. Glucose and Acrp30 treatment increased the size of neurospheres compared with the glucose treatment only. Scale bar: 200 *μ*m. Normal: control group; Glu (90 mM): 90 mM glucose treatment group; Glu (120 mM): 120 mM glucose treatment group; Acrp30: Acrp30 (30 *μ*g/ml) treatment group. (**b**) The graph showed the size of neurospheres in each group. In 90 and 120 mM glucose group, neurosphere size was attenuated in comparison with the normal control group. Under Acrp30 treatment, the neurospheres were increased compared with the glucose treatment only. *P<0.05, **P<0.001

We investigated whether neurosphere size of NSCs was changed by adiponectin at high glucose concentrations (Figure 7). Neurosphere size was measured using bright-field microscopy (Figure 7a). Neurosphere size was not altered by the adiponectin (Acrp30, 30 *μ*g/ml) treatment, and was similar to that in the control group. A treatment with 120 mM glucose reduced the neurosphere size compared with that in the control group. However, addition of Acrp30 (30 *μ*g/ml) and 120 mM glucose did not alter neurosphere size compared with that in the control group (Figures 7a and b). Especially, 120 mM glucose group showed significant differences with the control group (Figure 7b). Therefore, our data showed that adiponectin helps to maintain the normal size of NSC neurospheres at high glucose concentration.

### Adiponectin restored the reduced expression of AdipoR1 in the NSCs caused by high glucose concentrations

**Figure 8:**
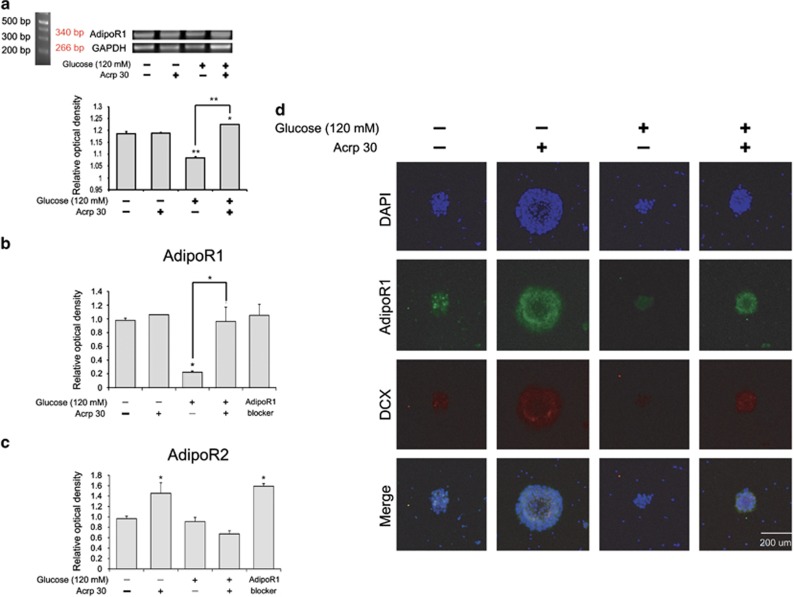
Measurement of AdipoR1 and AdipoR2 expression in the NSCs. The mRNA level of AdipoR1 (**a** and **b**) and AdipoR2 (**c**) was measured with reverse transcription PCR (**a**) and quantitative PCR (**b** and **c**). (**a** and **b**) Glucose treatment (120 mM) reduced the mRNA level of AdipoR1 compared with the control group. Glucose and Acrp30 treatments in combination increased the mRNA level of AdipoR1 compared with the glucose treatment. Data are expressed as mean±S.E.M., and each experiment included three repeats per condition. GAPDH was used as control gene. Differences were considered significant at *P<0.05 and **P<0.001. (**c**) Glucose treatment (120 mM) slightly reduced the mRNA level of AdipoR2 compared with the control group. Glucose and Acrp30 treatments in combination was not increased the mRNA level of AdipoR2 compared with the glucose treatment. Data are expressed as mean±S.E.M., and each experiment included three repeats per condition. GAPDH was used as control gene. Differences were considered significant at *P<0.05. (**d**) Immunohistochemical experiments revealed that the expression of AdipoR1 (green) was reduced in the glucose treatment group compared with that in the control group. Treatment with both glucose and Acrp30 increased AdipoR1 expression compared with the glucose treatment. Scale bar=200 *μ*m. 4',6-diamidino-2-phenylindole (DAPI): blue; adiponectin receptor 1 (AdipoR1): green; DCX: red

To examine AdipoR1 and AdipoR2 expression in the NSCs at high glucose concentrations, we conducted reverse transcription PCR (Figure 8a), quantitative PCR (Figures 8b and c) and immunohistochemical analysis (Figure 8d). At 120 mM glucose concentration, mRNA level of AdipoR1 in the NSCs was attenuated compared with that in the control group, whereas Acrp30 (30 *μ*g/ml) treatment restored the mRNA level of AdipoR1 reduced by high glucose concentrations (Figures 8a and b). At 120 mM glucose concentration, mRNA level of AdipoR2 in the NSCs was slightly attenuated compared with that in the control group, whereas Acrp30 (30 *μ*g/ml) treatment increased the mRNA level of AdipoR2. Acrp30 treatment did not largely increase the expression of AdipoR2 in high glucose concentrations (Figure 8c). In addition, we observed less AdipoR1 and DCX immunoreactivity in the NSCs exposed to high glucose concentration than in the control group (Figure 8d). However, the decreased immunoreactivity of AdipoR1 and DCX was enhanced by Acrp30 (30 μg/ml) treatment at high glucose concentrations (Figure 8d). Taken together, adiponectin may promote the expression of AdipoR1 and DCX in the NSCs exposed to high glucose concentrations.

### Adiponectin inhibits the pathways responsible for apoptosis in the NSCs at high glucose concentrations

**Figure 9:**
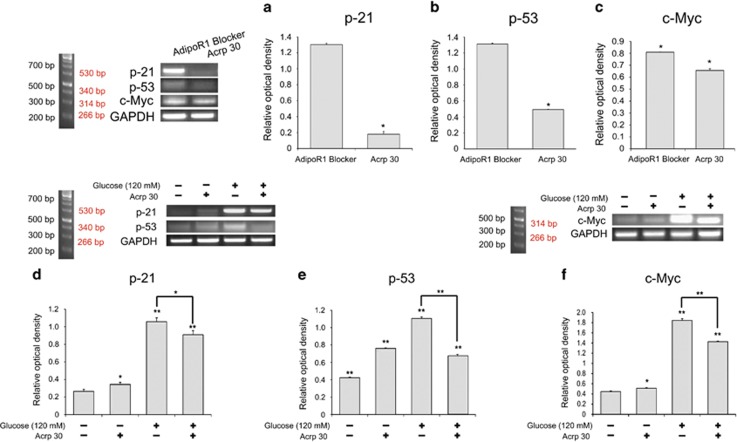
Measurement of mRNA levels in the NSCs using reverse transcription PCR. The mRNA levels of p21 (**a** and **d**), p53 (**b** and **e**), and c-Myc (**c** and **f**) in the NSCs were evaluated using reverse transcription PCR. Acrp30 (30 *μ*g/ml) treatment reduced the mRNA levels of p21 (**a**), p53 (**b**), and c-Myc (**c**) in the NSCs compared with the blocking of AdipoR1. The mRNA levels of p21 (**d**), p53 (**e**), and c-Myc (**f**) in the NSCs were reduced upon treatment with both glucose and Acrp30 (30 *μ*g/ml) compared with those treated with glucose. Data are expressed as mean±S.E.M., and each experiment included three repeats per condition. GAPDH was used as control gene. Differences were considered significant at *P<0.05 and **P<0.001

To investigate the effect of adiponectin on the apoptosis signaling pathways at high glucose concentrations, we measured the mRNA level of the apoptosis-related factors p21, p53, and c-Myc^51, 52, 53^ (Figure 9). Blocking AdipoR1 using AdipoR1 blocker compound increases the mRNA levels of p21 (Figure 9a), p53 (Figure 9b), and c-Myc (Figure 9c) in the NSCs compared with the Acrp30 (30 *μ*g/ml) treatment. The mRNA levels of p21, p53, and c-Myc in the NSCs were significantly reduced by treatment with both Acrp30 (30 *μ*g/ml) and glucose (120 mM) compared with treatment with glucose only (Figures 9d–f). We propose that adiponectin may inhibit the apoptosis pathways mediated by p21/p53 and c-Myc at high glucose concentrations.

### Adiponectin enhances neurogenesis and proliferation in the NSCs at high glucose concentrations

**Figure 10:**
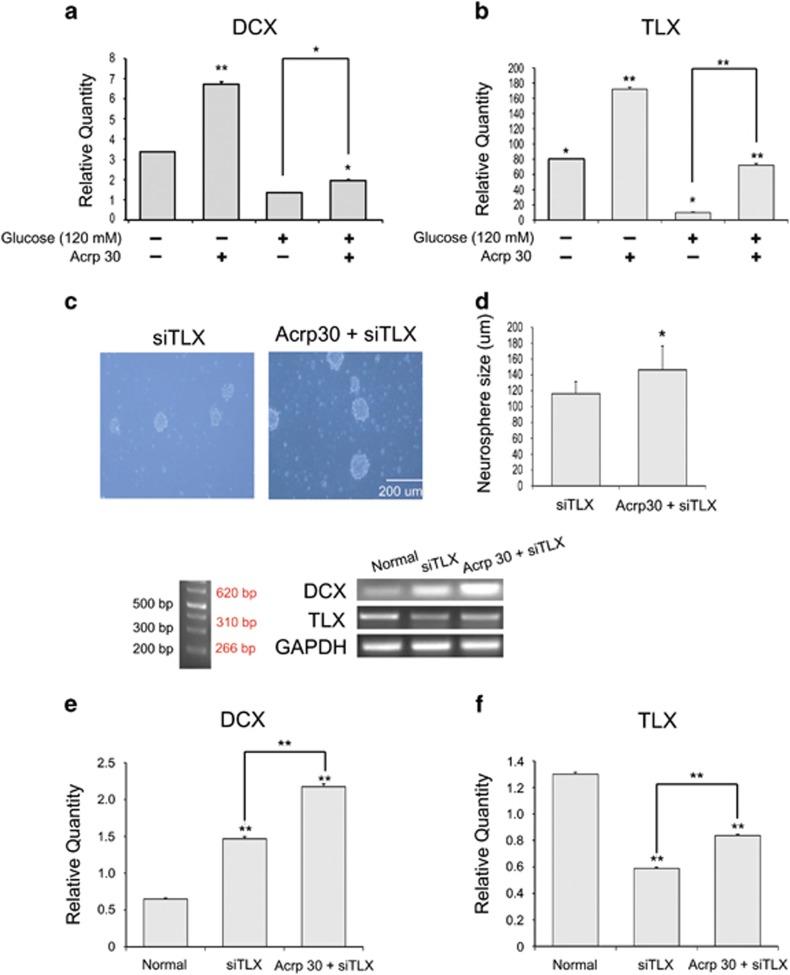
Measurement of DCX and TLX expression in the NSCs. The mRNA levels of DCX (**a**) and TLX (**b**) in the NSCs were evaluated using quantitative PCR. Treatment with both glucose and Acrp30 (30 *μ*g/ml) increased the mRNA levels of DCX (**a**) and TLX (**b**) compared with the glucose treatment only. (**c** and **d**) Neurosphere size was reduced by using siTLX, but Acrp30 (30 *μ*g/ml) treatment slightly increased neurosphere size. Data are expressed as mean±S.E.M., and each experiment included three repeats per condition. GAPDH was used as control gene. Differences were considered significant at *P<0.05. Blocking TLX significantly increased the mRNA levels of DCX (**e**) and TLX (**f**) in the NSCs treated with Acrp30 (30 *μ*g/ml). Data are expressed as mean±S.E.M., and each experiment included three repeats per condition. GAPDH was used as control gene. Differences were considered significant at *P<0.05 and **P<0.001

To study the effect of adiponectin on the neurogenesis and proliferation of NSCs at high glucose concentrations, we measured the mRNA levels of the neuronal cell factor DCX and the proliferation and neurogenesis-related factor TLX (Figure 10). Acrp30 (30 *μ*g/ml) increased the mRNA levels of DCX (Figure 10a) and TLX (Figure 10b) in the NSCs. In addition, under Acrp30 (30 μg/ml) with 120 mM glucose condition, the mRNA levels of DCX (Figure 10a) and TLX (Figure 10b) increased compared with the glucose treatment group (Figures 10a and b). We observed a decrease in neurosphere size by inhibiting TLX using siTLX, and an increase in neurosphere size by Acrp30 (30 *μ*g/ml) treatment (Figures 10c and d). Expressions of DCX and TLX in the NSCs were increased by TLX inhibition (Figures 10e and f). These data suggest that adiponectin might promote the neurogenesis related to DCX and TLX in the NSCs at high glucose concentrations.

## Discussion

Current research reports a positive correlation between DM2 and cognitive impairment in a prospective population-based study,^54^ and an accelerated progression from mild cognitive decline to dementia in the elderly patients with DM2 compared with the normal subjects.^55^ In addition, patients with diabetes (35%) or glucose intolerance (46%) have Alzheimer's disease in up to 80% of patients.^56^ Hyperglycemia is a main cause of neuronal damage, leading to neurodegeneration in the CNS.^57, 58, 59, 60^ Cognitive impairment in DM2 generally affects the brain regions responsible for learning and memory.^38, 61^ Patients with DM2-induced dementia have mitochondrial dysfunction and alterations in the neuronal synapses.^62, 63, 64^ Considering that the number of unhealthy and TUNEL-positive cells, which are considered as apoptotic,^65^ was increased in the brain of mice fed a HFD, we suggest that HFD leads to cellular damage in the brain. In addition, reduction of the postsynaptic protein PSD95, which is associated with memory,^66^ suggests that HFD impairs synaptic plasticity.

Memory dysfunction in type II diabetes mellitus^67^ triggers neuronal death and inhibits neurogenesis.^40, 68, 69^ In addition, HFD accelerates the progression of diabetes and inhibits neurogenesis in rodents.^70^ Patients with DM2 have decreased adiponectin plasma levels compared with normal subjects.^71^ In an animal study of DM2, decreased adiponectin level was considered the reason for the higher prevalence of insulin resistance.^72^ In the present study, we observed the decrease of AdipoR1 receptor ^13, 73, 74, 75, 76^ in various brain regions including the striatum, cortex, and hippocampus compared with the control group; however, we did not measure the plasma levels of adiponectin in the mice fed a HFD.

Recent studies demonstrated that HFD induces synaptic dysfunction^77^ and leads to neurotoxicity *in vivo*.^78^ Our results showed that the mRNA levels of p21, p53, and cMyc in the NSCs were increased at high glucose concentrations, whereas these effects were attenuated by adiponectin. The protein p53 triggers apoptosis,^79, 80^ inhibits self-renewal, induces the differentiation of embryonic stem cells,^81^ and blocks the reprogramming of progenitor cells into stem cells.^82, 83^ The activation of p53 and p21 indicates DNA damage in stem cells.^84, 85, 86, 87, 88^ In addition, the activation of p53/p21 signaling leads to cell arrest in the G_1_ and G_2_ phases of the cell cycle, and thus apoptosis.^89, 90^ Recent studies demonstrated that the increased apoptosis of NSCs through p53 and p21 inhibits proliferation.^84, 91^ Proliferation of bone marrow-derived stem cells is blocked by activating the cell cycle inhibitors p53 and p21.^92^ In an animal study investigating diabetes, the activation of p21 triggered the apoptosis of bone marrow-derived mesenchymal stem cells.^93^ In addition, p53 induces the transduction of c-Myc^94, 95^ and thus regulates various mechanisms such as apoptosis,^96^ cell growth,^97^ and self-renewal of embryonic stem cells.^98^ As the increase of p21, p53, and c-Myc induces apoptosis,^52, 99, 100, 101^ our results imply that adiponectin protects against cell death of NSCs during glucose-induced toxicity. In the present study, we showed that HFD attenuated the expression of DCX and TLX neurogenesis markers in the brain. The nuclear receptor TLX is required for neurogenesis in the subventricular zone.^102, 103^ Several studies reported that this orphan nuclear receptor increases the self-renewal of NSCs^104, 105, 106, 107^ and promotes the neurogenesis of neural precursor cells.^50, 108, 109, 110, 111^ TLX-positive cells are associated with learning and memory.^105^ Similarly, the neurogenesis-related protein DCX^112, 113^ is crucial for learning and memory,^114, 115, 116, 117^ and is involved in synaptic dysfunction.^118, 119, 120^ Our results showed that adiponectin increases the expression of TLX and DCX in the NSCs at high glucose concentrations, and that blocking AdipoR1 reduces TLX expression in the NSCs. Furthermore, the blockade of TLX did not change the size of neurospheres, and adiponectin treatment increased TLX expression.

In conclusion, our findings in the present study suggest the following. Considering our *in vivo* results, HFD might lead to the reduction of AdipoR1 that is related to cell survival,^121, 122, 123^ impaired synaptic plasticity,^124, 125, 126^ and attenuation of neurogenesis.^112, 127^ Considering our *in vitro* results, AdipoR1-mediated signaling might protect the NSCs against cell damage at high glucose concentrations, and might promote the self-renewal and neurogenesis of the NSCs at high glucose concentrations. The lack of clinical data is a limitation of this study. However, as our study has indicated the potential of adiponectin to alleviate hyperglycemia-induced neuropathogenesis, our results might spark further studies investigating adiponectin receptor signaling in the CNS.

## Materials and Methods

### Animal experiments

Male 3-week-old C57BL/6 mice (Orient, GyeongGi-Do, South Korea; http://www.orientbio.co.kr) were fed conventional chow or HFD; the latter was enriched in either fat (35.5% wt/wt; Bioserv, Frenchtown, NJ, USA) or fructose (60% wt/wt; Harlan Teklad, Madison, WI, USA) for 4 weeks. Then, animals on the HFD were injected once with streptozotocin (STZ; 100 mg/kg body weight; Sigma-Aldrich, St. Louis, MO, USA) intraperitoneally to induce partial insulin deficiency, and then the HFD was continued for an additional 4 weeks. The majority of mice in the STZ/HFD group exhibited hyperglycemia, insulin resistance, and glucose tolerance, as previously reported.^128^ The mice that were fed conventional chew (control group) were injected intraperitoneally with vehicle (0.05 mol/l citric acid, pH 4.5). To obtain their brains, mice were killed under ether anesthesia.

### Cresyl violet staining

After the mice were killed, their brains were fixed in 3.7% formaldehyde and immediately frozen. The brains were sectioned coronally at a thickness of 20 *μ*m, and the sections were sequentially incubated in xylene for 4 min, 100% alcohol for 5 min, 95% alcohol for 5 min, and 70% alcohol for 5 min. Samples were stained with cresyl violet (Sigma-Aldrich) for 5 min. After the staining, slides were dehydrated with 70% alcohol for 3 min, 95% alcohol for 3 min, 100% alcohol for 3 min, and xylene for 5 min. Sections were observed using a light microscope equipped with a digital camera (Olympus, Tokyo, Japan).

### TdT-mediated dUTP nick end labeling

Apoptotic cells were detected *in situ* using the Roche TUNEL kit (Roche, Mannheim, Germany) according to the manufacturer's protocol. The TUNEL assay was conducted to visualize the 3'-OH ends of DNA fragments in apoptotic cells. After xylene dewaxing, sections were rinsed three times in distilled water for 5 min, and they were washed in methanol containing 0.3% H_2_O_2_ at room temperature for 30 min to inhibit endogenous peroxidase activity. After rinsing in PBS three times at room temperature for 5 min, sections were treated with proteinase K at 37 °C for 6 min. Section were rinsed in PBS three times at room temperature for 3 min, were soaked in TdT buffer for 10 min, and incubated in 50 *μ*l TdT buffer containing TdT at 37 °C for 60 min in a moist chamber (Roche). After three rinsing steps in PBS at room temperature for 5 min, the sections were incubated in 50 *μ*l FITC (Roche) at 37 °C for 40 min. After three further rinses in PBS for 3 min, the brain sections were incubated in DAB (Roche) at room temperature for 3 min, and the signal was observed using a confocal microscope (Zeiss LSM 700, Carl Zeiss, Oberkochen, Germany).^129^

### Immunohistochemistry of the sections

Frozen brain sections, 5 *μ*m thick, were cut onto clean glass slides (Thermo Scientific, Waltham, MA, USA), air-dried, and fixed in cold acetone for 10 min at −20 °C. The slides were washed in Tris-buffered saline (TBS), and then incubated in 0.3% H_2_O_2_ in methanol to quench endogenous peroxidase activity. Followed by three washes in distilled water, the sections were blocked with 10% normal rabbit serum. To block nonspecific labeling, sections were incubated in 5% bovine serum albumin (BSA, Sigma-Aldrich) diluted in PBS for 30 min before the addition of primary and secondary antibodies. Sections were incubated with primary antibodies specific for AdipoR1 (1 : 100, Santa Cruz Biotechnology, Santa Cruz, CA, USA), DCX (1 : 100, Abcam, Cambridge, MA, USA), NeuN (1 : 100, Abcam), PSD95, 1 : 100, Millipore, Billerica, MA, USA), and orphan factor tailless (TLX, 1 : 100, Santa Cruz Biotechnology) for 24 h at 4 °C and then washed in PBS for 10 min. After three washes in 0.1% PBS with Tween-20 (PBST), sections were incubated for 2 h in the dark at room temperature with rhodamine-conjugated sheep anti-rabbit or fluorescein isothiocyanate (FITC)-conjugated sheep anti-mouse secondary antibodies that were diluted to 1 : 200 with 5% BSA in 0.1% PBST. After three washes in PBS, sections were incubated in 1 *μ*g/ml 4′,6-diamidino-2-phenylindole (Sigma-Aldrich) and 2 *μ*g/ml propidium iodide (Sigma-Aldrich) as a counterstain. Sections were observed using a confocal microscope (Zeiss LSM 700, Carl Zeiss, Oberkochen, Germany).

### NSC primary culture and drug treatment

Pregnant ICR mice were killed to obtain cortical primary NSCs according to a previously described method.^130^ The brains were extracted from E13.5 embryos, and placed in a Petri dish containing Hank's balanced salt solution (HBSS, Gibco, Grand Island, NY, USA). The cortices were dissected and washed 1–2 times with HBSS. To each piece of washed tissue, 5 ml HBSS was added, and the tissue was dissociated by pipetting up and down. Tissues were triturated by repeated passages through a fire-polished constricted Pasteur pipette. Dissociated tissues were allowed to settle for 3 min. Supernatants were transferred to a fresh tube, and were centrifuged at 1200 × *g* for 3 min. Pellets were resuspended in NSC basal media with a proliferation supplement (Stem Cell Technologies, Vancouver, BC, Canada), and 20 ng/ml epidermal growth factor (EGF, Invitrogen, Carlsbad, CA, USA). Live, Trypan blue-negative cells were counted. NSCs were plated on poly-D-ornithine (Sigma-Aldrich)-treated plastic dishes at a density of 2.5 × 10^4^ cells per ml. Cultures were maintained in a humidified atmosphere of 95% air and 5% CO_2_ at 37 °C. After 3 days *in vitro*, the cells proliferated and formed primary neurospheres. The primary neurospheres were harvested by centrifugation, and were dissociated to single cells using Accumax (Sigma-Aldrich). The single cells were seeded on culture plates coated with 0.001% poly-L-ornithine. Culture medium was replaced every 3 days. NSCs were used for experiments after 2–3 passages.^131^ NSCs were exposed to Acrp30 (30 *μ*g/ml, Sigma-Aldrich) for 4 days after 2–3 passages. Subsequently, cells were exposed to D-glucose The cells were also treated with the AdipoR1 blocking peptide (3 *μ*g/ml, GTX89569-PEP, GeneTex, Irvine, CA, USA) for 4 days of the experiment.^132^

### TLX silencing treatment

For the transfection, siRNA TLX (20 nM final concentration, Ambion, Austin, TX, USA) in Opti-MEM was mixed with Lipofectamine 2000 (Invitrogen) and incubated at room temperature for 10 min. The mixture was then added to the NSCs that incubated in the mixture for 72 h.

### Quantification of neurosphere size

Neurospheres were imaged using a bright-field inverted microscope (Olympus). The magnification (× 10) covered a significant area of each well from the 24-well plates. Ten non-overlapping fields were selected randomly from each well. All experiments were carried out 6 times.

### Reverse transcription PCR

To examine the expression of AdipoR1, c-Myc, p21, p53, DCX, and TLX in the NSCs, reverse transcription PCR was performed to measure their mRNA levels. Briefly, cells were lysed with Trizol reagent (Invitrogen), and total RNA was extracted according to the manufacturer's protocol. We synthesized cDNA from the mRNA. The following cycling conditions were used for the PCR: 10 min at 95 °C; 35 cycles of denaturing at 95 °C for 15 s, annealing for 30 s at 62 °C, elongation at 72 °C for 30 s; final extension for 10 min at 72 °C. PCR was performed using the following primers (5' to 3'): AdipoR1 (F): GAGCATCTTCCGCATTCATA, (R): AAGAGCCAGGAGAAGCTGAG; c-Myc (F): TCAAGAGGCGAACACACAAC, (R): GGCCTTTTCATTGTTTTCCA; p21 (F): AGTGTGCCGTTGTCTCTTCG, (R): ACACCAGAGTGCAAGACAGC; p-53 (F): CTGCCCTCAACAAGATGTTTTG, (R): CTATCTGAGCAGCGCTCATGG; TLX: (F): GGCTCTCTACTTCCGTGGACA, (R): GTCAGTATTCATGCCAGATACAGCCAGTG; DCX (F): AATCCCAACTGGTCTGTCAAC, (R): GTTTCCCTTCATGACTCGGCA; GAPDH (F): GGCATGGACTGTGGTCATGAG, (R): TGCACCACCAACTGCTTAGC. We examined the PCR products with electrophoresis using 1.5% agarose gels stained with ethidium bromide.

### Quantitative real-time PCR

To examine the amount of AdipoR1, DCX, and TLX mRNAs in the NSCs, we performed quantitative real-time PCR. Total RNA was extracted from the NSCs using the Trizol reagent (Invitrogen) according to the manufacturer's instructions. RNA was mixed with the One Step SYBR Prime Script TM RT-PCR Kit II (Takara, Otsu, Shiga, Japan) and the specific primers in 20 *μ*l total reaction volume. PCR was performed using the following primers (5′ to 3′); AdipoR1 (F): GAGCATCTTCCGCATTCATA, (R): AAGAGCCAGGAGAAGCTGAG; AdipoR2 (F): TGTTCGCCACCCCTCAGTAT, (R): AATGATTCCACTCAGGCCCA, TLX (F): GGCTCTCTACTTCCGTGGACA, (R): GTCAGTATTCATGCCAGATACAGCCAGTG; DCX (F): AATCCCAACTGGTCTGTCAAC, (R): GTTTCCCTTCATGACTCGGCA; GAPDH (F): GGCATGGACTGTGGTCATGAG, (R): TGCACCACCAACTGCTTAGC. The PCR was performed at 42 °C for 5 min, 95 °C for 10 s, followed by 40 cycles of 95 °C for 15 s, 60 °C for 34 s, and 65 °C for 15 s. Quantitative PCR was performed with the ABI prism 7500 Real-Time PCR System (Life Technologies, Grand Island, NY, USA), and we analyzed the C_t_ values using relative quantification.^133^ Data were normalized to the reference housekeeping gene *GAPDH*. The ΔC_t_ values of the treated cells were compared with those of the untreated cells.

### Western blot analysis

Cells were washed rapidly with ice-cold PBS, scraped, and collected. Cell pellets were lysed with ice-cold RIPA buffer (Sigma-Aldrich). Lysates were centrifuged at 13 000 r.p.m. for 1 h at 4 °C to produce whole-cell extracts. Protein content was quantified using the BCA kit (Pierce, Rockford, IL, USA). Proteins (40 *μ*g) were separated on a 10% sodium dodecyl sulfate-polyacrylamide gel and then transferred onto a polyvinylidene difluoride membrane. After blocking with 5% BSA in Tris-buffered saline/Tween (TBS-T; 20 nM Tris (pH 7.2), 150 mM NaCl, and 0.1% Tween-20) for 1 h at room temperature, immunoblots were incubated overnight at 4 °C with the primary antibodies specific for PSD95 (1 : 1000, Millipore), DCX (1 : 1000, Abcam), TLX (1 : 1000, Santa Cruz Biotechnology), AdipoR1 (1 : 1000, Santa Cruz Biotechnology), AdipoR2 (1 : 1000, Santa Cruz Biotechnology), and *β*-actin (1 : 2000, Cell Signaling Technology, Danvers, MA, USA). Next, the blots were incubated with horseradish peroxidase-linked anti-mouse and anti-rabbit IgG antibodies (Abcam) for 1 h at room temperature. Chemiluminescence signal was developed using an ECL kit (Invitrogen).

### Immunocytochemistry of the NSCs

NSCs were washed three times with PBS, and were permeabilized for 30 min. NSCs were incubated with the primary antibodies overnight at 4 °C. The following primary antibodies were used: anti-goat AdipoR1 (1 : 200, Santa Cruz Biotechnology) and anti-rabbit DCX (1 : 200, Abcam). The primary antibody was then removed, and the cells were washed three times for 3 min with PBS. Cells were incubated with FITC-conjugated donkey anti-goat IgG (1 : 200, Jackson Immunoresearch, West Grove, PA, USA), and rhodamine-conjugated goat anti-rabbit IgG (1 : 200, Jackson Immunoresearch) for 2 h at room temperature. NSCs were washed again three times for 3 min with PBS. NSCs were then counterstained with 1 *μ*g/ml 4',6-diamidino-2-phenylindole (DAPI, 1 : 200, Invitrogen) for 10 min at room temperature. Cells were imaged using a Zeiss LSM 700 confocal microscope (Carl Zeiss, Thornwood, NY, USA).

### Statistical analysis

Statistical analyses were carried out using the SPSS 18.0 software (IBM Corporation, Armonk, NY, USA). All data are expressed as mean±S.E.M. Significant intergroup differences were determined by one-way analysis of variance (ANOVA) followed by the Bonferroni *post hoc* multiple comparison test. Each experiment included three replicates per condition. Differences were considered significant at *P<0.05 and **P<0.001.

## Abbreviations

AdipoR1: adiponectin receptor 1
AdipoR2: adiponectin receptor 2
HFD: high-fat diet
NSCs: neural stem cells
DCX: doublecortin

## References

[bib1] Scherer PE Williams S Fogliano M Baldini G Lodish HF A novel serum protein similar to C1q, produced exclusively in adipocytes J Biol Chem 1995 270 26746 26749 10.1074/jbc.270.45.26746 7592907

[bib2] Maeda K Okubo K Shimomura I Funahashi T Matsuzawa Y Matsubara K cDNA cloning and expression of a novel adipose specific collagen-like factor, apM1 (AdiPose Most abundant Gene transcript 1) Biochem Biophys Res Commun 1996 221 286 289 10.1006/bbrc.1996.0587 8619847

[bib3] Fruebis J Tsao TS Javorschi S Ebbets-Reed D Erickson MR Yen FT Proteolytic cleavage product of 30-kDa adipocyte complement-related protein increases fatty acid oxidation in muscle and causes weight loss in mice Proc Natl Acad Sci USA 2001 98 2005 2010 11172066 10.1073/pnas.041591798 PMC29372

[bib4] Waki H Yamauchi T Kamon J Kita S Ito Y Hada Y Generation of globular fragment of adiponectin by leukocyte elastase secreted by monocytic cell line THP-1 Endocrinology 2005 146 790 796 15528304 10.1210/en.2004-1096

[bib5] Tsao TS Tomas E Murrey HE Hug C Lee DH Ruderman NB Role of disulfide bonds in Acrp30/adiponectin structure and signaling specificity. Different oligomers activate different signal transduction pathways J Biol Chem 2003 278 50810 50817 14522956 10.1074/jbc.M309469200

[bib6] Waki H Yamauchi T Kamon J Ito Y Uchida S Kita S Impaired multimerization of human adiponectin mutants associated with diabetes. Molecular structure and multimer formation of adiponectin J Biol Chem 2003 278 40352 40363 12878598 10.1074/jbc.M300365200

[bib7] Qi Y Takahashi N Hileman SM Patel HR Berg AH Pajvani UB Adiponectin acts in the brain to decrease body weight Nat Med 2004 10 524 529 15077108 10.1038/nm1029

[bib8] Kubota N Yano W Kubota T Yamauchi T Itoh S Kumagai H Adiponectin stimulates AMP-activated protein kinase in the hypothalamus and increases food intake Cell Metab 2007 6 55 68 17618856 10.1016/j.cmet.2007.06.003

[bib9] Neumeier M Weigert J Buettner R Wanninger J Schaffler A Muller AM Detection of adiponectin in cerebrospinal fluid in humans Am J Physiol Endocrinol Metab 2007 293 E965 E969 17623750 10.1152/ajpendo.00119.2007

[bib10] Kusminski CM McTernan PG Schraw T Kos K O'Hare JP Ahima R Adiponectin complexes in human cerebrospinal fluid: distinct complex distribution from serum Diabetologia 2007 50 634 642 17242917 10.1007/s00125-006-0577-9

[bib11] Ebinuma H Miida T Yamauchi T Hada Y Hara K Kubota N Improved ELISA for selective measurement of adiponectin multimers and identification of adiponectin in human cerebrospinal fluid Clin Chem 2007 53 1541 1544 17599956 10.1373/clinchem.2007.085654

[bib12] Yamauchi T Kamon J Ito Y Tsuchida A Yokomizo T Kita S Cloning of adiponectin receptors that mediate antidiabetic metabolic effects Nature 2003 423 762 769 12802337 10.1038/nature01705

[bib13] Kadowaki T Yamauchi T Adiponectin and adiponectin receptors Endocr Rev 2005 26 439 451 15897298 10.1210/er.2005-0005

[bib14] Staiger H Kaltenbach S Staiger K Stefan N Fritsche A Guirguis A Expression of adiponectin receptor mRNA in human skeletal muscle cells is related to *in vivo* parameters of glucose and lipid metabolism Diabetes 2004 53 2195 2201 15331527 10.2337/diabetes.53.9.2195

[bib15] Neumeier M Weigert J Schaffler A Weiss T Kirchner S Laberer S Regulation of adiponectin receptor 1 in human hepatocytes by agonists of nuclear receptors Biochem Biophys Res Commun 2005 334 924 929 16023994 10.1016/j.bbrc.2005.06.187

[bib16] Palanivel R Fang X Park M Eguchi M Pallan S De Girolamo S Globular and full-length forms of adiponectin mediate specific changes in glucose and fatty acid uptake and metabolism in cardiomyocytes Cardiovasc Res 2007 75 148 157 17499232 10.1016/j.cardiores.2007.04.011

[bib17] Berner HS Lyngstadaas SP Spahr A Monjo M Thommesen L Drevon CA Adiponectin and its receptors are expressed in bone-forming cells Bone 2004 35 842 849 15454091 10.1016/j.bone.2004.06.008

[bib18] Fry M Smith PM Hoyda TD Duncan M Ahima RS Sharkey KA Area postrema neurons are modulated by the adipocyte hormone adiponectin J Neurosci 2006 26 9695 9702 16988040 10.1523/JNEUROSCI.2014-06.2006 PMC6674457

[bib19] Nawrocki AR Rajala MW Tomas E Pajvani UB Saha AK Trumbauer ME Mice lacking adiponectin show decreased hepatic insulin sensitivity and reduced responsiveness to peroxisome proliferator-activated receptor gamma agonists J Biol Chem 2006 281 2654 2660 16326714 10.1074/jbc.M505311200

[bib20] Maeda N Shimomura I Kishida K Nishizawa H Matsuda M Nagaretani H Diet-induced insulin resistance in mice lacking adiponectin/ACRP30 Nat Med 2002 8 731 737 12068289 10.1038/nm724

[bib21] Kubota N Terauchi Y Yamauchi T Kubota T Moroi M Matsui J Disruption of adiponectin causes insulin resistance and neointimal formation J Biol Chem 2002 277 25863 25866 12032136 10.1074/jbc.C200251200

[bib22] Yamauchi T Kamon J Waki H Terauchi Y Kubota N Hara K The fat-derived hormone adiponectin reverses insulin resistance associated with both lipoatrophy and obesity Nat Med 2001 7 941 946 11479627 10.1038/90984

[bib23] Combs TP Pajvani UB Berg AH Lin Y Jelicks LA Laplante M A transgenic mouse with a deletion in the collagenous domain of adiponectin displays elevated circulating adiponectin and improved insulin sensitivity Endocrinology 2004 145 367 383 14576179 10.1210/en.2003-1068

[bib24] Yamauchi Y Hayashi M Abe-Dohmae S Yokoyama S Apolipoprotein A-I activates protein kinase C alpha signaling to phosphorylate and stabilize ATP binding cassette transporter A1 for the high density lipoprotein assembly J Biol Chem 2003 278 47890 47897 12952980 10.1074/jbc.M306258200

[bib25] Ohashi K Shibata R Murohara T Ouchi N Role of anti-inflammatory adipokines in obesity-related diseases Trends Endocrinol Metab 2014 25 348 355 24746980 10.1016/j.tem.2014.03.009

[bib26] Ouchi N Walsh K Adiponectin as an anti-inflammatory factor Clin Chim Acta 2007 380 24 30 17343838 10.1016/j.cca.2007.01.026 PMC2755046

[bib27] Lopresti AL Drummond PD Obesity and psychiatric disorders: commonalities in dysregulated biological pathways and their implications for treatment Prog Neuropsychopharmacol Biol Psychiatry 2013 45 92 99 23685202 10.1016/j.pnpbp.2013.05.005

[bib28] Zhang D Guo M Zhang W Lu XY Adiponectin stimulates proliferation of adult hippocampal neural stem/progenitor cells through activation of p38 mitogen-activated protein kinase (p38MAPK)/glycogen synthase kinase 3beta (GSK-3beta)/beta-catenin signaling cascade J Biol Chem 2011 286 44913 44920 22039048 10.1074/jbc.M111.310052 PMC3247954

[bib29] Kukull WA Higdon R Bowen JD McCormick WC Teri L Schellenberg GD Dementia and Alzheimer disease incidence: a prospective cohort study Arch Neurol 2002 59 1737 1746 12433261 10.1001/archneur.59.11.1737

[bib30] Marioni RE Deary IJ Strachan MW Lowe GD Rumley A Murray GD Blood rheology and cognition in the Edinburgh Type 2 Diabetes Study Age Ageing 2010 39 354 359 20197283 10.1093/ageing/afq021

[bib31] Crane PK Walker R Hubbard RA Li G Nathan DM Zheng H Glucose levels and risk of dementia N Engl J Med 2013 369 540 548 23924004 10.1056/NEJMoa1215740 PMC3955123

[bib32] Whitmer RA Karter AJ Yaffe K Quesenberry CP Jr Selby JV Hypoglycemic episodes and risk of dementia in older patients with type 2 diabetes mellitus JAMA 2009 301 1565 1572 19366776 10.1001/jama.2009.460 PMC2782622

[bib33] Arvanitakis Z Wilson RS Bienias JL Evans DA Bennett DA Diabetes mellitus and risk of Alzheimer disease and decline in cognitive function Arch Neurol 2004 61 661 666 15148141 10.1001/archneur.61.5.661

[bib34] Li J Chen X Xiao W Ma W Li T Huang J Mitochondria-targeted antioxidant peptide SS31 attenuates high glucose-induced injury on human retinal endothelial cells Biochem Biophys Res Commun 2011 404 349 356 21134355 10.1016/j.bbrc.2010.11.122

[bib35] Schiff M Loublier S Coulibaly A Benit P de Baulny HO Rustin P Mitochondria and diabetes mellitus: untangling a conflictive relationship J Inherit Metab Dis 2009 32 684 698 19821144 10.1007/s10545-009-1263-0

[bib36] Herlein JA Fink BD Sivitz WI Superoxide production by mitochondria of insulin-sensitive tissues: mechanistic differences and effect of early diabetes Metabolism 2010 59 247 257 19765776 10.1016/j.metabol.2009.07.021 PMC2813404

[bib37] Toth C Martinez J Zochodne DW RAGE, diabetes, and the nervous system Curr Mol Med 2007 7 766 776 18331235 10.2174/156652407783220705

[bib38] Kodl CT Seaquist ER Cognitive dysfunction and diabetes mellitus Endocr Rev 2008 29 494 511 18436709 10.1210/er.2007-0034 PMC2528851

[bib39] Pluchino S Muzio L Imitola J Deleidi M Alfaro-Cervello C Salani G Persistent inflammation alters the function of the endogenous brain stem cell compartment Brain 2008 131 2564 2578 18757884 10.1093/brain/awn198 PMC2570715

[bib40] Ekdahl CT Kokaia Z Lindvall O Brain inflammation and adult neurogenesis: the dual role of microglia Neuroscience 2009 158 1021 1029 18662748 10.1016/j.neuroscience.2008.06.052

[bib41] Kim SJ Son TG Park HR Park M Kim MS Kim HS Curcumin stimulates proliferation of embryonic neural progenitor cells and neurogenesis in the adult hippocampus J Biol Chem 2008 283 14497 14505 18362141 10.1074/jbc.M708373200 PMC2386914

[bib42] Zhang WJ Tan YF Yue JT Vranic M Wojtowicz JM Impairment of hippocampal neurogenesis in streptozotocin-treated diabetic rats Acta Neurol Scand 2008 117 205 210 17854417 10.1111/j.1600-0404.2007.00928.x

[bib43] Beauquis J Saravia F Coulaud J Roig P Dardenne M Homo-Delarche F Prominently decreased hippocampal neurogenesis in a spontaneous model of type 1 diabetes, the nonobese diabetic mouse Exp Neurol 2008 210 359 367 18190910 10.1016/j.expneurol.2007.11.009

[bib44] Stranahan AM Arumugam TV Cutler RG Lee K Egan JM Mattson MP Diabetes impairs hippocampal function through glucocorticoid-mediated effects on new and mature neurons Nat Neurosci 2008 11 309 317 18278039 10.1038/nn2055 PMC2927988

[bib45] Puglielli L Tanzi RE Kovacs DM Alzheimer's disease: the cholesterol connection Nat Neurosci 2003 6 345 351 12658281 10.1038/nn0403-345

[bib46] Taghibiglou C Bradley CA Gaertner T Li Y Wang Y Wang YT Mechanisms involved in cholesterol-induced neuronal insulin resistance Neuropharmacology 2009 57 268 276 19523477 10.1016/j.neuropharm.2009.05.013

[bib47] Grasl-Kraupp B Ruttkay-Nedecky B Koudelka H Bukowska K Bursch W Schulte-Hermann R *In situ* detection of fragmented DNA (TUNEL assay) fails to discriminate among apoptosis, necrosis, and autolytic cell death: a cautionary note Hepatology 1995 21 1465 1468 7737654 10.1002/hep.1840210534

[bib48] Murai K Qu Q Sun G Ye P Li W Asuelime G Nuclear receptor TLX stimulates hippocampal neurogenesis and enhances learning and memory in a transgenic mouse model Proc Natl Acad Sci USA 2014 111 9115 9120 24927526 10.1073/pnas.1406779111 PMC4078800

[bib49] Li S Sun G Murai K Ye P Shi Y Characterization of TLX expression in neural stem cells and progenitor cells in adult brains PLoS One 2012 7 e43324 22952666 10.1371/journal.pone.0043324 PMC3431389

[bib50] Islam MM Zhang CL TLX: a master regulator for neural stem cell maintenance and neurogenesis Biochim Biophys Acta 2015 1849 210 216 24930777 10.1016/j.bbagrm.2014.06.001 PMC4265312

[bib51] Farazi PA Zeisberg M Glickman J Zhang Y Kalluri R DePinho RA Chronic bile duct injury associated with fibrotic matrix microenvironment provokes cholangiocarcinoma in p53-deficient mice Cancer Res 2006 66 6622 6627 16818635 10.1158/0008-5472.CAN-05-4609

[bib52] Miao W Liu X Wang H Fan Y Lian S Yang X p53 upregulated modulator of apoptosis sensitizes drug-resistant U251 glioblastoma stem cells to temozolomide through enhanced apoptosis Mol Med Rep 2015 11 4165 4173 25625235 10.3892/mmr.2015.3255 PMC4394929

[bib53] Zhu X Zhang K Wang Q Chen S Gou Y Cui Y Cisplatin-mediated c-myc overexpression and cytochrome c (cyt c) release result in the up-regulation of the death receptors DR4 and DR5 and the activation of caspase 3 and caspase 9, likely responsible for the TRAIL-sensitizing effect of cisplatin Med Oncol 2015 32 588 10.1007/s12032-015-0588-9 25796504

[bib54] Yaffe K Falvey CM Hamilton N Harris TB Simonsick EM Strotmeyer ES Association between hypoglycemia and dementia in a biracial cohort of older adults with diabetes mellitus JAMA Intern Med 2013 173 1300 1306 23753199 10.1001/jamainternmed.2013.6176 PMC4041621

[bib55] Alagiakrishnan K Sclater A Psychiatric disorders presenting in the elderly with type 2 diabetes mellitus Am J Geriatr Psychiatry 2012 20 645 652 21989315 10.1097/JGP.0b013e31823038db

[bib56] Janson J Laedtke T Parisi JE O'Brien P Petersen RC Butler PC Increased risk of type 2 diabetes in Alzheimer disease Diabetes 2004 53 474 481 14747300 10.2337/diabetes.53.2.474

[bib57] Wang HL Chou AH Wu AS Chen SY Weng YH Kao YC PARK6 PINK1 mutants are defective in maintaining mitochondrial membrane potential and inhibiting ROS formation of substantia nigra dopaminergic neurons Biochim Biophys Acta 2011 1812 674 684 21421046 10.1016/j.bbadis.2011.03.007

[bib58] Toth C Brussee V Cheng C Zochodne DW Diabetes mellitus and the sensory neuron J Neuropathol Exp Neurol 2004 63 561 573 15217085 10.1093/jnen/63.6.561

[bib59] Biessels GJ Deary IJ Ryan CM Cognition and diabetes: a lifespan perspective Lancet Neurol 2008 7 184 190 18207116 10.1016/S1474-4422(08)70021-8

[bib60] Messier C Impact of impaired glucose tolerance and type 2 diabetes on cognitive aging Neurobiol Aging 2005 26 26 30 16236384 10.1016/j.neurobiolaging.2005.09.014

[bib61] Convit A Links between cognitive impairment in insulin resistance: an explanatory model Neurobiol Aging 2005 26 31 35 16246463 10.1016/j.neurobiolaging.2005.09.018

[bib62] Mattson MP Pathways towards and away from Alzheimer's disease Nature 2004 430 631 639 15295589 10.1038/nature02621 PMC3091392

[bib63] Das F Dey N Venkatesan B Kasinath BS Ghosh-Choudhury N Choudhury GG High glucose upregulation of early-onset Parkinson's disease protein DJ-1 integrates the PRAS40/TORC1 axis to mesangial cell hypertrophy Cell Signal 2011 23 1311 1319 21426932 10.1016/j.cellsig.2011.03.012 PMC3104472

[bib64] Matsuzaki T Sasaki K Tanizaki Y Hata J Fujimi K Matsui Y Insulin resistance is associated with the pathology of Alzheimer disease: the Hisayama study Neurology 2010 75 764 770 20739649 10.1212/WNL.0b013e3181eee25f

[bib65] van Lookeren Campagne M Gill R Ultrastructural morphological changes are not characteristic of apoptotic cell death following focal cerebral ischaemia in the rat Neurosci Lett 1996 213 111 114 8858621 10.1016/0304-3940(96)12839-1

[bib66] Lee CC Huang CC Wu MY Hsu KS Insulin stimulates postsynaptic density-95 protein translation via the phosphoinositide 3-kinase-Akt-mammalian target of rapamycin signaling pathway J Biol Chem 2005 280 18543 18550 15755733 10.1074/jbc.M414112200

[bib67] MacLullich AM Seckl JR Diabetes and cognitive decline: are steroids the missing link Cell Metab 2008 7 286 287 18396133 10.1016/j.cmet.2008.03.012

[bib68] Das S Basu A Inflammation: a new candidate in modulating adult neurogenesis J Neurosci Res 2008 86 1199 1208 18058947 10.1002/jnr.21585

[bib69] Wiltrout C Lang B Yan Y Dempsey RJ Vemuganti R Repairing brain after stroke: a review on post-ischemic neurogenesis Neurochem Int 2007 50 1028 1041 17531349 10.1016/j.neuint.2007.04.011

[bib70] Lindqvist A Mohapel P Bouter B Frielingsdorf H Pizzo D Brundin P High-fat diet impairs hippocampal neurogenesis in male rats Eur J Neurol 2006 13 1385 1388 17116226 10.1111/j.1468-1331.2006.01500.x

[bib71] Li S Shin HJ Ding EL van Dam RM Adiponectin levels and risk of type 2 diabetes: a systematic review and meta-analysis JAMA 2009 302 179 188 19584347 10.1001/jama.2009.976

[bib72] Wakshlag JJ Struble AM Levine CB Bushey JJ Laflamme DP Long GM The effects of weight loss on adipokines and markers of inflammation in dogs Br J Nutr 2011 106 S11 S14 22005402 10.1017/S0007114511000560

[bib73] Thundyil J Pavlovski D Sobey CG Arumugam TV Adiponectin receptor signalling in the brain Br J Pharmacol 2012 165 313 327 21718299 10.1111/j.1476-5381.2011.01560.x PMC3268187

[bib74] Elmquist JK Ahima RS Maratos-Flier E Flier JS Saper CB Leptin activates neurons in ventrobasal hypothalamus and brainstem Endocrinology 1997 138 839 842 9003024 10.1210/endo.138.2.5033

[bib75] Murphy KT Schwartz GJ Nguyen NL Mendez JM Ryu V Bartness TJ Leptin-sensitive sensory nerves innervate white fat Am J Physiol Endocrinol Metab 2013 304 E1338 E1347 23612999 10.1152/ajpendo.00021.2013 PMC3680695

[bib76] Sachot C Rummel C Bristow AF Luheshi GN The role of the vagus nerve in mediating the long-term anorectic effects of leptin J Neuroendocrinol 2007 19 250 261 17355316 10.1111/j.1365-2826.2006.01528.x

[bib77] Arnold SE Lucki I Brookshire BR Carlson GC Browne CA Kazi H High fat diet produces brain insulin resistance, synaptodendritic abnormalities and altered behavior in mice Neurobiol Dis 2014 67 79 87 24686304 10.1016/j.nbd.2014.03.011 PMC4083060

[bib78] De Felice FG Vieira MN Bomfim TR Decker H Velasco PT Lambert MP Protection of synapses against Alzheimer's-linked toxins: insulin signaling prevents the pathogenic binding of Abeta oligomers Proc Natl Acad Sci USA 2009 106 1971 1976 19188609 10.1073/pnas.0809158106 PMC2634809

[bib79] Cicalese A Bonizzi G Pasi CE Faretta M Ronzoni S Giulini B The tumor suppressor p53 regulates polarity of self-renewing divisions in mammary stem cells Cell 2009 138 1083 1095 19766563 10.1016/j.cell.2009.06.048

[bib80] Morgado-Palacin L Llanos S Serrano M Ribosomal stress induces L11- and p53-dependent apoptosis in mouse pluripotent stem cells Cell Cycle 2012 11 503 510 22262176 10.4161/cc.11.3.19002

[bib81] Lin T Chao C Saito S Mazur SJ Murphy ME Appella E p53 induces differentiation of mouse embryonic stem cells by suppressing Nanog expression Nat Cell Biol 2005 7 165 171 15619621 10.1038/ncb1211

[bib82] Kawamura T Suzuki J Wang YV Menendez S Morera LB Raya A Linking the p53 tumour suppressor pathway to somatic cell reprogramming Nature 2009 460 1140 1144 19668186 10.1038/nature08311 PMC2735889

[bib83] Hong H Takahashi K Ichisaka T Aoi T Kanagawa O Nakagawa M Suppression of induced pluripotent stem cell generation by the p53-p21 pathway Nature 2009 460 1132 1135 19668191 10.1038/nature08235 PMC2917235

[bib84] Insinga A Cicalese A Faretta M Gallo B Albano L Ronzoni S DNA damage in stem cells activates p21, inhibits p53, and induces symmetric self-renewing divisions Proc Natl Acad Sci USA 2013 110 3931 3936 23417300 10.1073/pnas.1213394110 PMC3593901

[bib85] Sperka T Wang J Rudolph KL DNA damage checkpoints in stem cells, ageing and cancer Nat Rev Mol Cell Biol 2012 13 579 590 22914294 10.1038/nrm3420

[bib86] el-Deiry WS Tokino T Velculescu VE Levy DB Parsons R Trent JM WAF1, a potential mediator of p53 tumor suppression Cell 1993 75 817 825 8242752 10.1016/0092-8674(93)90500-p

[bib87] Harper JW Adami GR Wei N Keyomarsi K Elledge SJ The p21 Cdk-interacting protein Cip1 is a potent inhibitor of G1 cyclin-dependent kinases Cell 1993 75 805 816 8242751 10.1016/0092-8674(93)90499-g

[bib88] Zhang DY Wang HJ Tan YZ Wnt/beta-catenin signaling induces the aging of mesenchymal stem cells through the DNA damage response and the p53/p21 pathway PLoS One 2011 6 e21397 21712954 10.1371/journal.pone.0021397 PMC3119703

[bib89] Boonstra J Post JA Molecular events associated with reactive oxygen species and cell cycle progression in mammalian cells Gene 2004 337 1 13 15276197 10.1016/j.gene.2004.04.032

[bib90] Valente LJ Gray DH Michalak EM Pinon-Hofbauer J Egle A Scott CL p53 efficiently suppresses tumor development in the complete absence of its cell-cycle inhibitory and proapoptotic effectors p21, Puma, and Noxa Cell Rep 2013 3 1339 1345 23665218 10.1016/j.celrep.2013.04.012

[bib91] Bian X McAllister-Lucas LM Shao F Schumacher KR Feng Z Porter AG NF-kappa B activation mediates doxorubicin-induced cell death in N-type neuroblastoma cells J Biol Chem 2001 276 48921 48929 11679590 10.1074/jbc.M108674200

[bib92] Chen K Perez-Stable C D'Ippolito G Schiller PC Roos BA Howard GA Human bone marrow-derived stem cell proliferation is inhibited by hepatocyte growth factor via increasing the cell cycle inhibitors p53, p21 and p27 Bone 2011 49 1194 1204 21907315 10.1016/j.bone.2011.08.023

[bib93] Gu Z Jiang J Xia Y Yue X Yan M Tao T p21 is associated with the proliferation and apoptosis of bone marrow-derived mesenchymal stem cells from non-obese diabetic mice Exp Clin Endocrinol Diabetes 2013 121 607 613 24277484 10.1055/s-0033-1354380

[bib94] Motohara T Masuko S Ishimoto T Yae T Onishi N Muraguchi T Transient depletion of p53 followed by transduction of c-Myc and K-Ras converts ovarian stem-like cells into tumor-initiating cells Carcinogenesis 2011 32 1597 1606 21828057 10.1093/carcin/bgr183

[bib95] Akita H Marquardt JU Durkin ME Kitade M Seo D Conner EA MYC activates stem-like cell potential in hepatocarcinoma by a p53-dependent mechanism Cancer Res 2014 74 5903 5913 25189530 10.1158/0008-5472.CAN-14-0527 PMC4199878

[bib96] Zheng H Ying H Yan H Kimmelman AC Hiller DJ Chen AJ Pten and p53 converge on c-Myc to control differentiation, self-renewal, and transformation of normal and neoplastic stem cells in glioblastoma Cold Spring Harb Symp Quant Biol 2008 73 427 437 19150964 10.1101/sqb.2008.73.047

[bib97] Dang CV c-Myc target genes involved in cell growth, apoptosis, and metabolism Mol Cell Biol 1999 19 1 11 9858526 10.1128/mcb.19.1.1 PMC83860

[bib98] Smith KN Lim JM Wells L Dalton S Myc orchestrates a regulatory network required for the establishment and maintenance of pluripotency Cell Cycle 2011 10 592 597 21293186 10.4161/cc.10.4.14792 PMC3173999

[bib99] Ye M Zhang J Zhang J Miao Q Yao L Zhang J Curcumin promotes apoptosis by activating the p53-miR-192-5p/215-XIAP pathway in non-small cell lung cancer Cancer Lett 2015 357 196 205 25444916 10.1016/j.canlet.2014.11.028

[bib100] Chang X Lu W Dou T Wang X Lou D Sun X Paraquat inhibits cell viability via enhanced oxidative stress and apoptosis in human neural progenitor cells Chem Biol Interact 2013 206 248 255 24060684 10.1016/j.cbi.2013.09.010

[bib101] Insinga A Cicalese A Pelicci PG DNA damage response in adult stem cells Blood Cells Mol Dis 2014 52 147 151 24484934 10.1016/j.bcmd.2013.12.005

[bib102] Liu HK Belz T Bock D Takacs A Wu H Lichter P The nuclear receptor tailless is required for neurogenesis in the adult subventricular zone Genes Dev 2008 22 2473 2478 18794344 10.1101/gad.479308 PMC2546695

[bib103] Monaghan AP Grau E Bock D Schutz G The mouse homolog of the orphan nuclear receptor tailless is expressed in the developing forebrain Development 1995 121 839 853 7720587 10.1242/dev.121.3.839

[bib104] Shi Y Chichung Lie D Taupin P Nakashima K Ray J Yu RT Expression and function of orphan nuclear receptor TLX in adult neural stem cells Nature 2004 427 78 83 14702088 10.1038/nature02211

[bib105] Zhang CL Zou Y He W Gage FH Evans RM A role for adult TLX-positive neural stem cells in learning and behaviour Nature 2008 451 1004 1007 18235445 10.1038/nature06562

[bib106] Sun G Yu RT Evans RM Shi Y Orphan nuclear receptor TLX recruits histone deacetylases to repress transcription and regulate neural stem cell proliferation Proc Natl Acad Sci USA 2007 104 15282 15287 17873065 10.1073/pnas.0704089104 PMC2000559

[bib107] Li W Sun G Yang S Qu Q Nakashima K Shi Y Nuclear receptor TLX regulates cell cycle progression in neural stem cells of the developing brain Mol Endocrinol 2008 22 56 64 17901127 10.1210/me.2007-0290 PMC2194628

[bib108] Elmi M Matsumoto Y Zeng ZJ Lakshminarasimhan P Yang W Uemura A TLX activates MASH1 for induction of neuronal lineage commitment of adult hippocampal neuroprogenitors Mol Cell Neurosci 2010 45 121 131 20599619 10.1016/j.mcn.2010.06.003

[bib109] Green HF Nolan YM Unlocking mechanisms in interleukin-1beta-induced changes in hippocampal neurogenesis—a role for GSK-3beta and TLX Transl Psychiatry 2012 2 e194 23168994 10.1038/tp.2012.117 PMC3565766

[bib110] Roy K Kuznicki K Wu Q Sun Z Bock D Schutz G The Tlx gene regulates the timing of neurogenesis in the cortex J Neurosci 2004 24 8333 8345 15385616 10.1523/JNEUROSCI.1148-04.2004 PMC2740800

[bib111] Zhang C Wu H Zhu X Wang Y Guo J Role of transcription factors in neurogenesis after cerebral ischemia Rev Neurosci 2011 22 457 465 21692687 10.1515/RNS.2011.034

[bib112] Couillard-Despres S Winner B Schaubeck S Aigner R Vroemen M Weidner N Doublecortin expression levels in adult brain reflect neurogenesis Eur J Neurosci 2005 21 1 14 15654838 10.1111/j.1460-9568.2004.03813.x

[bib113] Vellema M Hertel M Urbanus SL Van der Linden A Gahr M Evaluating the predictive value of doublecortin as a marker for adult neurogenesis in canaries (Serinus canaria) J Comp Neurol 2014 522 1299 1315 24115109 10.1002/cne.23476

[bib114] Karl C Couillard-Despres S Prang P Munding M Kilb W Brigadski T Neuronal precursor-specific activity of a human doublecortin regulatory sequence J Neurochem 2005 92 264 282 15663475 10.1111/j.1471-4159.2004.02879.x

[bib115] Vukovic J Borlikova GG Ruitenberg MJ Robinson GJ Sullivan RK Walker TL Immature doublecortin-positive hippocampal neurons are important for learning but not for remembering J Neurosci 2013 33 6603 6613 23575857 10.1523/JNEUROSCI.3064-12.2013 PMC6619068

[bib116] Hernandez-Rabaza V Llorens-Martin M Velazquez-Sanchez C Ferragud A Arcusa A Gumus HG Inhibition of adult hippocampal neurogenesis disrupts contextual learning but spares spatial working memory, long-term conditional rule retention and spatial reversal Neuroscience 2009 159 59 68 19138728 10.1016/j.neuroscience.2008.11.054

[bib117] Siwak-Tapp CT Head E Muggenburg BA Milgram NW Cotman CW Neurogenesis decreases with age in the canine hippocampus and correlates with cognitive function Neurobiol Learn Mem 2007 88 249 259 17587610 10.1016/j.nlm.2007.05.001 PMC2173881

[bib118] Wang S Scott BW Wojtowicz JM Heterogenous properties of dentate granule neurons in the adult rat J Neurobiol 2000 42 248 257 10640331

[bib119] Cheng X Li Y Huang Y Feng X Feng G Xiong ZQ Pulse labeling and long-term tracing of newborn neurons in the adult subgranular zone Cell Res 2011 21 338 349 20938464 10.1038/cr.2010.141 PMC3193432

[bib120] Ge S Yang CH Hsu KS Ming GL Song H A critical period for enhanced synaptic plasticity in newly generated neurons of the adult brain Neuron 2007 54 559 566 17521569 10.1016/j.neuron.2007.05.002 PMC2040308

[bib121] Chou IP Lin YY Ding ST Chen CY Adiponectin receptor 1 enhances fatty acid metabolism and cell survival in palmitate-treated HepG2 cells through the PI3 K/AKT pathway Eur J Nutr 2014 53 907 917 24129500 10.1007/s00394-013-0594-7

[bib122] Chan KH Lam KS Cheng OY Kwan JS Ho PW Cheng KK Adiponectin is protective against oxidative stress induced cytotoxicity in amyloid-beta neurotoxicity PLoS One 2012 7 e52354 23300647 10.1371/journal.pone.0052354 PMC3531475

[bib123] Nepal S Kim MJ Subedi A Lee ES Yong CS Kim JA Globular adiponectin inhibits ethanol-induced apoptosis in HepG2 cells through heme oxygenase-1 induction Biochem Pharmacol 2012 84 974 983 22842631 10.1016/j.bcp.2012.07.019

[bib124] Chao HW Tsai LY Lu YL Lin PY Huang WH Chou HJ Deletion of CPEB3 enhances hippocampus-dependent memory via increasing expressions of PSD95 and NMDA receptors J Neurosci 2013 33 17008 17022 24155305 10.1523/JNEUROSCI.3043-13.2013 PMC6618447

[bib125] Sultana R Banks WA Butterfield DA Decreased levels of PSD95 and two associated proteins and increased levels of BCl2 and caspase 3 in hippocampus from subjects with amnestic mild cognitive impairment: insights into their potential roles for loss of synapses and memory, accumulation of Abeta, and neurodegeneration in a prodromal stage of Alzheimer's disease J Neurosci Res 2010 88 469 477 19774677 10.1002/jnr.22227 PMC2843415

[bib126] Nyffeler M Zhang WN Feldon J Knuesel I Differential expression of PSD proteins in age-related spatial learning impairments Neurobiol Aging 2007 28 143 155 16386336 10.1016/j.neurobiolaging.2005.11.003

[bib127] Bekiari C Giannakopoulou A Siskos N Grivas I Tsingotjidou A Michaloudi H Neurogenesis in the septal and temporal part of the adult rat dentate gyrus Hippocampus 2014 25 511 523 25394554 10.1002/hipo.22388

[bib128] Luo J Quan J Tsai J Hobensack CK Sullivan C Hector R Nongenetic mouse models of non-insulin-dependent diabetes mellitus Metabolism 1998 47 663 668 9627363 10.1016/s0026-0495(98)90027-0

[bib129] de Torres C Munell F Ferrer I Reventos J Macaya A Identification of necrotic cell death by the TUNEL assay in the hypoxic-ischemic neonatal rat brain Neurosci Lett 1997 230 1 4 9259449 10.1016/s0304-3940(97)00445-x

[bib130] Gritti A Parati EA Cova L Frolichsthal P Galli R Wanke E Multipotential stem cells from the adult mouse brain proliferate and self-renew in response to basic fibroblast growth factor J Neurosci 1996 16 1091 1100 8558238 10.1523/JNEUROSCI.16-03-01091.1996 PMC6578802

[bib131] Moriya T Horie N Mitome M Shinohara K Melatonin influences the proliferative and differentiative activity of neural stem cells J Pineal Res 2007 42 411 418 17439558 10.1111/j.1600-079X.2007.00435.x

[bib132] Grossini E Prodam F Walker GE Sigaudo L Farruggio S Bellofatto K Effect of monomeric adiponectin on cardiac function and perfusion in anesthetized pig J Endocrinol 2014 222 137 149 24860147 10.1530/JOE-14-0170

[bib133] Popivanova BK Kitamura K Wu Y Kondo T Kagaya T Kaneko S Blocking TNF-alpha in mice reduces colorectal carcinogenesis associated with chronic colitis J Clin Invest 2008 118 560 570 18219394 10.1172/JCI32453 PMC2213370

